# Calciphylaxis: An Unusual and Silent Usher for an Underlying Carcinoma

**DOI:** 10.7759/cureus.48363

**Published:** 2023-11-06

**Authors:** Aviraag Vijaya Prakash, Madhan Srinivasan Kumar, Jessica Veulens

**Affiliations:** 1 Internal Medicine, Saint Vincent Hospital, Worcester, USA

**Keywords:** alcohol-related liver disease, calciphylaxis complications, hepato cellular carcinoma, non-uremic calciphylaxis diagnosis, non uremic calciphylaxis

## Abstract

Calciphylaxis is an uncommon but serious and potentially life-threatening painful condition that is characterized by cutaneous vascular calcification and ischemia. It is typically seen in patients with end-stage renal disease but has been known to occur less commonly in patients with normal renal function. A rare but established etiology of non-uremic calciphylaxis is malignancy. We have thus far been successful in documenting an association between calciphylaxis and certain solid tumor types. The objective of this clinical case study is to better define a possible association between calciphylaxis and underlying malignancy.

## Introduction

Calciphylaxis is an uncommon condition characterized by microvascular calcification and thrombosis, which subsequently leads to cutaneous tissue necrosis [1]. Calciphylaxis has been long since established as a complication of end-stage renal disease (ESRD), especially in patients on dialysis. It has been thought that the predisposition in renal disease patients is because there is severe dysregulation of calcium-phosphate metabolism due to the ubiquitous nature of mineral bone abnormalities along with the use of pro-calcification treatments such as calcium salts in patients with kidney disease [2]. However, over the years, calciphylaxis has presented in patients with normal kidney function, such as autoimmune conditions [3], POEMS (polyneuropathy, organomegaly, endocrinopathy, monoclonal protein, and skin changes) syndrome [4], and most recently coronavirus disease 2019 (COVID-19) [5] and rarely in some underlying malignancies [6,7]. We present a case of calciphylaxis without any familiar risk factors, and we describe how his cutaneous presentation eventually led us to find an underlying hepatocellular carcinoma.

## Case presentation

A 65-year-old gentleman with a past medical history significant for liver cirrhosis secondary to chronic alcohol use, depression, and generalized anxiety disorder presented to the hospital with complaints of worsening bilateral lower limb discoloration which was associated with swelling, pain, and small ulcers. He reported that these symptoms began two months prior to presentation and denied any trauma, insect bite, change in food habits, or exposure to sick individuals preceding the development of symptoms. He reported about a 15-pound unintentional weight loss over a span of eight months. He denied any history of fevers, rash, or ulcers on other parts of the body, joint pain, abdominal pain, and easy bruising. Pertinent social history included daily consumption of three to four glasses of alcohol and a remote history of intravenous (IV) drug use. He also reported using over-the-counter cream for lower limb discoloration with no significant improvement. His other routine medications were quetiapine, mirtazapine, and citalopram, but he was noted to be non-compliant. At the time of presentation, he noted immense pain in both legs which was significantly limiting his ambulation.

**Figure 1:**
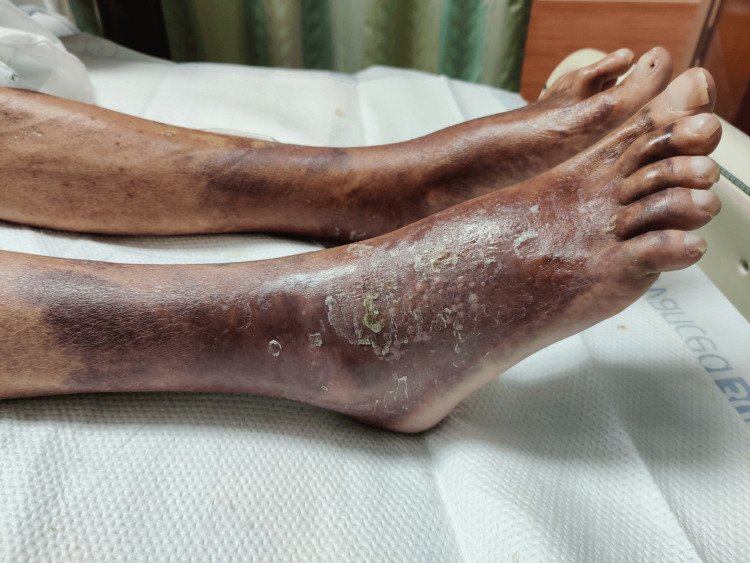
Calciphylaxis of the lower extremity involving the feet and extending above the ankle

On physical examination, his vitals were stable, but he appeared emaciated. He was found to have violaceous discoloration of both legs extending 4-6 inches above his ankles (Figure 1). There were also minor areas of scaling and ulceration with no associated discharge. The affected areas were exquisitely tender but had no appreciable warmth on palpation. The rest of his physical exam was grossly unremarkable. On initial laboratory evaluation, his complete blood count, basic metabolic panel, and urine analysis were normal. ESR was mildly elevated at 16 mm/hr with a normal C-reactive protein (CRP) of 4.61. Calcium was noted to be 8.5 mg/dL and phosphorus 3 mg/dL. His Liver function tests were deranged with total bilirubin elevated at 2.6 mg/dL, direct bilirubin at 0.8 mg/dL, aspartate aminotransferase (AST), and alanine aminotransferase (ALT) elevated to 88 U/L and 69 U/L respectively, albumin 3.0 g/dL, and INR 1.2. Infectious diseases work up for Anaplasma, Babesia, Ehrlichia, Hepatitis panel, and HIV were negative. Autoimmune work up including antinuclear antibody, anti-cytoplasmic antibody, and complement levels were normal. Testing for nutritional deficiencies of zinc, niacin, and pyridoxine was non-contributory. Bilateral lower limb venous duplex did not reveal any deep vein thrombosis. Based on our testing and examination we were able to successfully rule out cellulitis, erythroderma, tick-borne conditions, deep vein thrombosis, and vasculitis.

**Figure 2:**
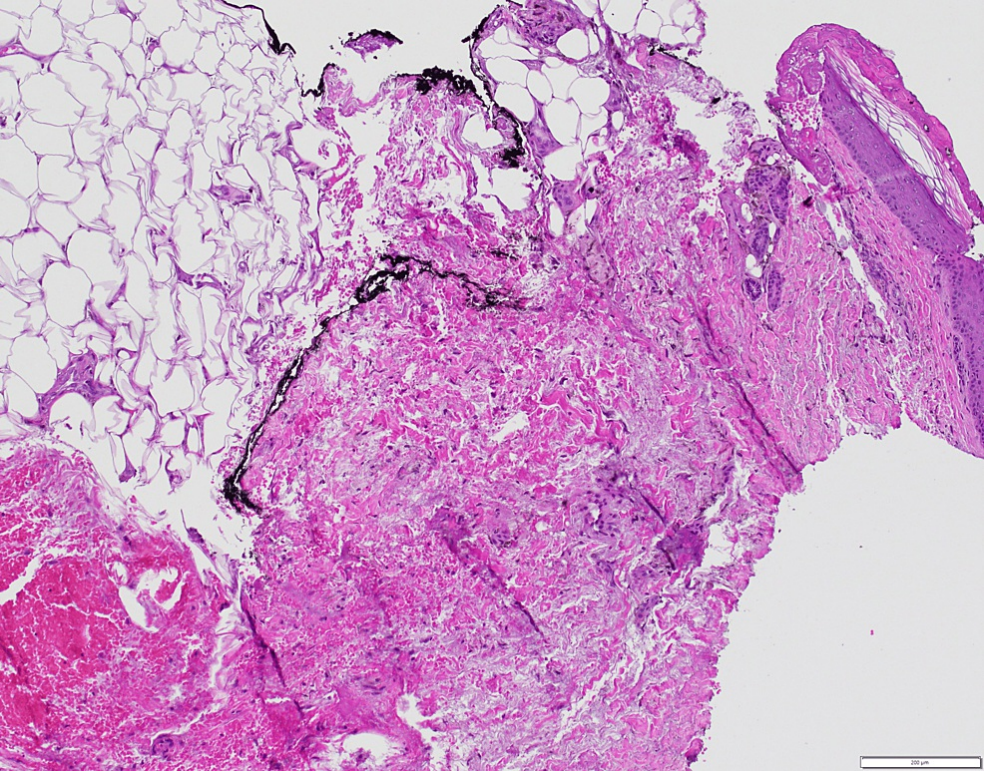
Punch biopsy of the skin of the lower extremity From left to right in the image, are demonstrated fat necrosis, haemorrhage, and chronic inflammatory changes.

With an initial suspicion of vasculitis, a short course of oral prednisone along with topical betamethasone was tried with no improvement. For his persistent pain, he was given ibuprofen with adequate response. For further management, a punch biopsy of the affected skin was obtained (Figure 2).

**Figure 3:**
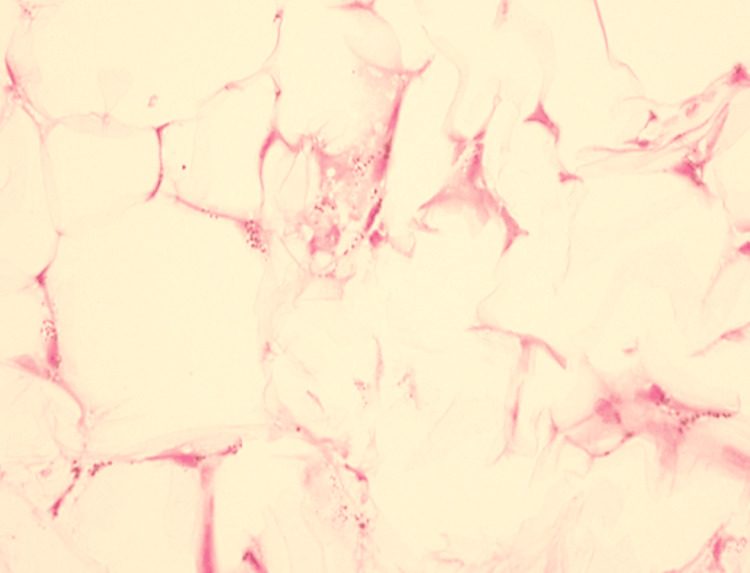
Stippled calcification highlighted on special staining of Von Kossa

A Von Kossa staining was performed which showed subcutaneous fat necrosis, and hemorrhage with perivascular calcification, consistent with calciphylaxis (Figure 3).

**Figure 4:**
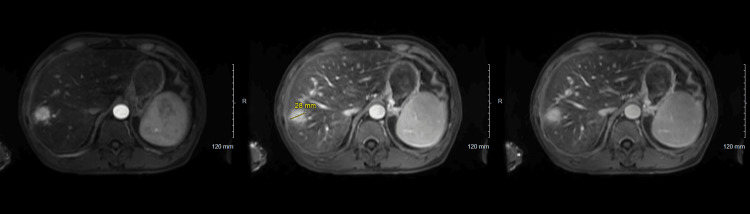
Contrast imaging MRI MRI images demonstrate a mass highly suspicious for hepatocellular carcinoma in the right hepatic lobe. Moving from left to right are the images of three post-contrast phases: arterial phase, portal phase, and vasculo-Kupffer phase.

Due to the history of weight loss and liver cirrhosis, we obtained an abdominal CT that showed a 3.8 cm right hepatic lobe mass, and due to suspicion of hepatocellular carcinoma (HCC), an MRI with contrast was obtained. MRI revealed findings highly suggestive of hepatocellular carcinoma, with splenomegaly and lymphadenopathy in the porta and celiac regions (Figure 4). Unfortunately, after discussing the imaging findings with the patient, he denied further investigations and opted out of further treatment.

## Discussion

Calciphylaxis is a serious medical condition with an insidious onset, with a male-to-female ratio of incidence being 12:1, and commonly affects the skin of the legs, followed by toes, fingers, abdomen, buttocks, and penis [8]. It has been frequently associated with end-stage renal disease; however, there have been sporadic cases of non-uremic calciphylaxis. A systemic review in 2008, described 36 cases of non-uremic calciphylaxis, out of which eight cases were reported malignancies such as metastatic breast cancer, cholangiocarcinoma, osteosclerotic myeloma, malignant melanoma, and chronic myelomonocytic leukemia [9].

Our patient had no significant past medical history preceding this presentation apart from alcoholic cirrhosis and mood disorders for which he was non-compliant with medications. The drugs that have been identified to cause calciphylaxis are calcium supplements, calcium-based phosphate binders, vitamin D, warfarin, parenteral iron, corticosteroids, and trauma from subcutaneous administration of heparin and insulin [10-12]. Thus, there was no identifiable risk factor at the time that could have predisposed him to develop calciphylaxis apart from the newly diagnosed hepatic malignancy. To our knowledge, there have been no such cases of calciphylaxis reported thus far.

Although MRI is currently the second-line imaging modality to diagnose hepatocellular carcinoma (HCC), it boasts an impressive sensitivity of 84.4% and a specificity of 93.8% for diagnosing HCC, based on 34 prior studies involving 4841 participants [13]. Moreover, MRI is the gold standard imaging modality to identify HCC and is effective in differentiating neoplasm from benign lesions. Extracellular contrast agent MRI has been accepted as a diagnostic modality for liver transplantation without tissue biopsy in clinical practice for HCC less than 2 cm [14].

Calciphylaxis is typically a tissue diagnosis and histopathological findings include calcification, micro-thrombi, and fibro-intimal hyperplasia of dermal and subcutaneous arteries and arterioles with subsequent ischemia and septal panniculitis [2]. Currently, it has been accepted that in vascular calcification, pathogenesis begins with the transformation of vascular smooth muscle cells (VSMCs) into osteoblast-like phenotypes [15]. This occurs via a complex interaction between the vascular smooth muscle cells with uremic toxins and reactive oxygen species, coupled with a decrease in matrix G1a protein (MGP), a potent inhibitor of calcification [16]. It has been established that there is an increase in reactive oxygen species in almost all cancer types [17] and it has also been found that there is a reduced expression of matrix G1a protein messenger RNA in malignancy [18]. In addition to vascular calcification and subintimal fibrosis, the role of hypercoagulability is integral in the pathogenesis of calciphylaxis, demonstrated by findings of thrombosis in histopathology [11]. Inflammatory cytokines, such as tumor necrosis factor (TNF)-alpha, interleukin (IL)-1, and IL-6 are known to decrease the anti-thrombotic receptor expression of thrombomodulin, protein C/S, and vascular heparin-like molecules, thus promoting thrombosis [19]. Neutrophil extracellular traps, which are externalized decondensed chromatin from the azurophilic granules have also been implicated in sterile inflammation leading to thrombosis in malignant and autoimmune conditions [20].

Unfortunately, there is no approved therapy for the management of calciphylaxis with treatment being primarily focused on preventing complications and symptom control. Pain should be adequately managed and disruptions in the skin should be promptly managed with appropriate wound care and if necessary, antibiotics. Several experimental options such as hyperbaric oxygen, bisphosphonates, vitamin K, and low-dose tissue plasminogen activators are being studied with variable results [21]. Sodium thiosulphate has been found to be beneficial in managing both uremic and nonuremic calciphylaxis [22].

## Conclusions

Besides the potentially lethal prognosis of the underlying malignancies that cause calciphylaxis, the added complications of calciphylaxis, such as superinfection leading to sepsis, are potentially fatal. In conclusion, calciphylaxis should be considered as a differential in patients having risk factors for malignancy along with typical symptom spectrum who present with a painful, non-healing cutaneous condition. Also, in the appropriate clinical setting, it is important to screen for cancers in patients diagnosed with calciphylaxis.

## References

[1] Rick J, Strowd L, Pasieka HB (2022). Calciphylaxis: Part I. Diagnosis and pathology. J Am Acad Dermatol.

[2] Nigwekar SU, Kroshinsky D, Nazarian RM (2015). Calciphylaxis: risk factors, diagnosis, and treatment. Am J Kidney Dis.

[3] Tsuchiya K, Endo C, Kondo A (2021). A case of non-uremic calciphylaxis associated with systemic lupus erythematosus and antiphospholipid syndrome. J Dermatol.

[4] Heck D, Mergen M, Ganner A, Pelisek J, Mader I, Weiller C, Niesen WD (2014). POEMS syndrome, calciphylaxis and focal segmental glomerulosclerosis - VEGF as a possible link. BMC Neurol.

[5] Rotman JA, Dean KE, Magro C, Nuovo G, Bartolotta RJ (2020). Concomitant calciphylaxis and COVID-19 associated thrombotic retiform purpura. Skeletal Radiol.

[6] Afridi SM, Raja A, Zhou X, Jain A (2019). Calciphylaxis due to metastatic well-differentiated neuroendocrine carcinoma. BMJ Case Rep.

[7] Hawkes JE, Karavan M, Bowen AR, Summers EM (2014). Nonuraemic calciphylaxis and pancreatic cancer: a previously unreported association. Br J Dermatol.

[8] Zhou Y, Chen Y, Yin G, Xie Q (2023). Calciphylaxis and its co-occurrence with connective tissue diseases. Int Wound J.

[9] Nigwekar SU, Wolf M, Sterns RH, Hix JK (2008). Calciphylaxis from nonuremic causes: a systematic review. Clin J Am Soc Nephrol.

[10] Fine A, Zacharias J (2002). Calciphylaxis is usually non-ulcerating: risk factors, outcome and therapy. Kidney Int.

[11] Weenig RH, Sewell LD, Davis MD, McCarthy JT, Pittelkow MR (2007). Calciphylaxis: natural history, risk factor analysis, and outcome. J Am Acad Dermatol.

[12] Farah M, Crawford RI, Levin A, Chan Yan C (2011). Calciphylaxis in the current era: emerging 'ironic' features?. Nephrol Dial Transplant.

[13] Nadarevic T, Colli A, Giljaca V (2022). Magnetic resonance imaging for the diagnosis of hepatocellular carcinoma in adults with chronic liver disease. Cochrane Database Syst Rev.

[14] Roberts LR, Sirlin CB, Zaiem F (2018). Imaging for the diagnosis of hepatocellular carcinoma: a systematic review and meta-analysis. Hepatology.

[15] Moe SM, Chen NX (2008). Mechanisms of vascular calcification in chronic kidney disease. J Am Soc Nephrol.

[16] Sowers KM, Hayden MR (2010). Calcific uremic arteriolopathy: pathophysiology, reactive oxygen species and therapeutic approaches. Oxid Med Cell Longev.

[17] Liou GY, Storz P (2010). Reactive oxygen species in cancer. Free Radic Res.

[18] Fan C, Sheu D, Fan H, Hsu K, Allen Chang C, Chan E (2001). Down-regulation of matrix Gla protein messenger RNA in human colorectal adenocarcinomas. Cancer Lett.

[19] Saghazadeh A, Hafizi S, Rezaei N (2015). Inflammation in venous thromboembolism: cause or consequence?. Int Immunopharmacol.

[20] Herre M, Cedervall J, Mackman N, Olsson AK (2023). Neutrophil extracellular traps in the pathology of cancer and other inflammatory diseases. Physiol Rev.

[21] Rick J, Rrapi R, Chand S (2022). Calciphylaxis: treatment and outlook-CME part II. J Am Acad Dermatol.

[22] Ning MS, Dahir KM, Castellanos EH, McGirt LY (2013). Sodium thiosulfate in the treatment of non-uremic calciphylaxis. J Dermatol.

